# Association of prescription opioid use on mortality and hospital length of stay in the intensive care unit

**DOI:** 10.1371/journal.pone.0250320

**Published:** 2021-04-22

**Authors:** Nicole Hardy, Fatima Zeba, Anaelia Ovalle, Alicia Yanac, Christelle Nzugang-Noutonsi, Mike Abadier, Anais Ovalle, Abdullah Chahin

**Affiliations:** 1 Department of Biostatistics, Brown University School of Public Health, Providence, RI, United States of America; 2 Department of Medicine, Kent Hospital/Brown University, Warwick, RI, United States of America; 3 Department of Computer Science, University of California Los Angeles, Los Angeles, California, United States of America; 4 Department of Medicine, Leadership Preventive Medicine Dartmouth-Hitchcock Medical Center, Lebanon, NH, United States of America; 5 Alpert School of Medicine, Brown University, Providence, RI, United States of America; BronxCare Health System, Affiliated with Icahn School of Medicine at Mount Sinai, NY, USA, UNITED STATES

## Abstract

**Objective:**

Several studies show that chronic opioid dependence leads to higher in-hospital mortality, increased risk of hospital readmissions, and worse outcomes in trauma cases. However, the association of outpatient prescription opioid use on morbidity and mortality has not been adequately evaluated in a critical care setting. The purpose of this study was to determine if there is an association between chronic opioid use and mortality after an ICU admission.

**Design:**

A single-center, longitudinal retrospective cohort study of all Intensive Care Unit (ICU) patients admitted to a tertiary-care academic medical center from 2001 to 2012 using the MIMIC-III database.

**Setting:**

Medical Information Mart for Intensive Care III database based in the United States.

**Patients:**

Adult patients 18 years and older were included. Exclusion criteria comprised of patients who expired during their hospital stay or presented with overdose; patients with cancer, anoxic brain injury, non-prescription opioid use; or if an accurate medication reconciliation was unable to be obtained. Patients prescribed chronic opioids were compared with those who had not been prescribed opioids in the outpatient setting.

**Interventions:**

None.

**Measurements and main results:**

The final sample included a total of 22,385 patients, with 2,621 (11.7%) in the opioid group and 19,764 (88.3%) in the control group. After proceeding with bivariate analyses, statistically significant and clinically relevant differences were identified between opioid and non-opioid users in sex, length of hospital stay, and comorbidities. Opioid use was associated with increased mortality in both the 30-day and 1-year windows with a respective odds ratios of 1.81 (95% CI, 1.63–2.01; p<0.001) and 1.88 (95% CI, 1.77–1.99; p<0.001), respectively.

**Conclusions:**

Chronic opioid usage was associated with increased hospital length of stay and increased mortality at both 30 days and 1 year after ICU admission. Knowledge of this will help providers make better choices in patient care and have a more informed risk-benefits discussion when prescribing opioids for chronic usage.

## Introduction

Opioid dependence is a significant contributor to the global burden of disease [1]. The opioid crisis in the US has been apparent and a target of many interventions. While the overall national opioid prescribing rate declined from 2012 to 2018 (the lowest rate is still 51.4 prescriptions per 100 persons in 2018), the total number has never fallen below 168 million opioid prescriptions [2]. Furthermore, recent trends show a marked increase in outpatient use of chronic opioid therapy for chronic non-cancer pain without decreases in reported pain, raising concerns about the efficacy and risk to benefit ratio of opioids in this population [3–7].

Studies show that chronic opioid dependence leads to higher in-hospital mortality, increased risk of hospital readmissions, and worse outcomes in trauma cases [8–10]. However, the association of outpatient prescription opioid use on morbidity and mortality has not been adequately evaluated in a critical care setting. The purpose of this study was to determine whether a statistically significant association exists between chronic opioid use and mortality after an ICU stay. We also explore the association of illness severity on admission, change in illness severity after admission, comorbidities and demographics on this outcome. To do this we developed three models for each of the outcomes: length of hospital stay, 30-day mortality and 1-year mortality. Knowledge of this will help providers make better choices in patient care and have a more informed risk-benefits discussion when prescribing opioids for chronic usage.

## Materials and methods

We performed a single-center, longitudinal retrospective cohort study of all Intensive Care Unit (ICU) patients admitted to a tertiary-care academic medical center from 2001 to 2012 using the MIMIC-III database [11].

**Fig 1:**
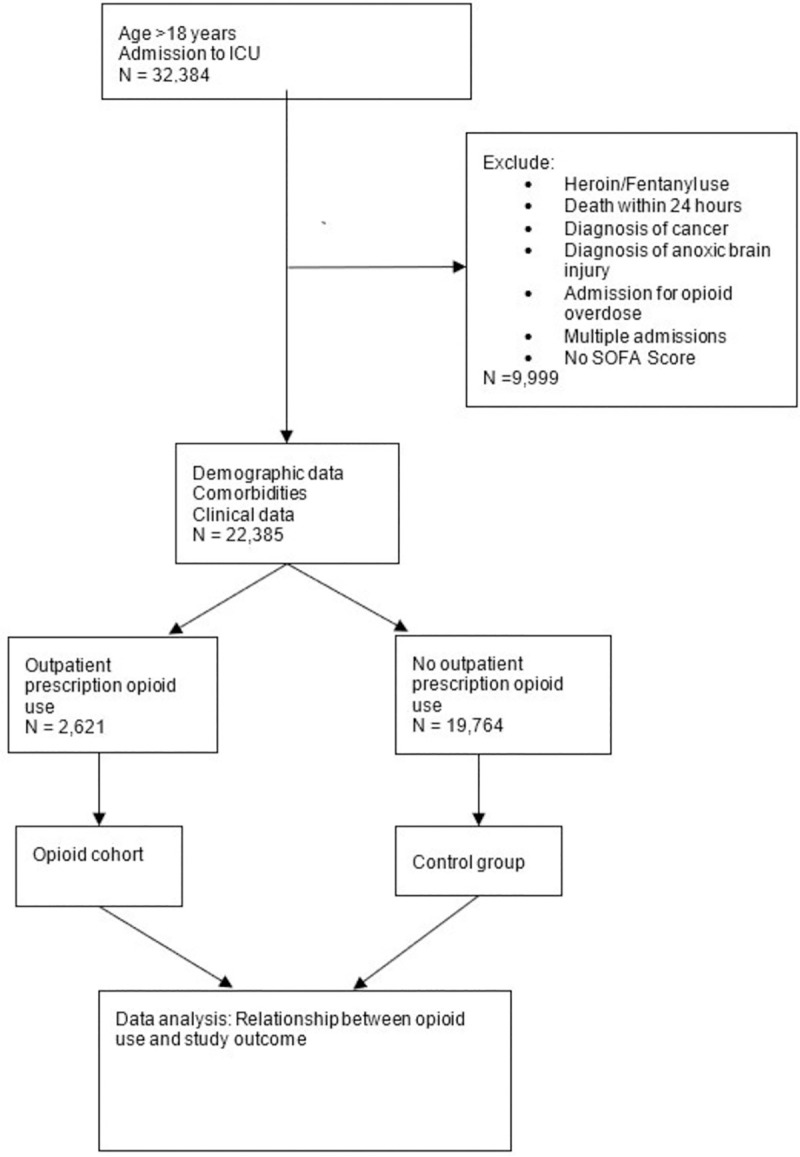
Flow chart of data extraction and analysis.

MIMIC III is a widely used, reliable, de-identified electronic database created by MIT using EMR information from patients at the Beth Israel Deaconess Medical Center. It is open access and available for research purposes. Informed consent and ethical approval were not required due to the anonymization. Patients age 18 years and older were included in the study, and we defined chronic opioid use by the presence of an outpatient opioid prescription on admission to the ICU. Exclusion criteria included patients who expired during their hospital stay or presented with overdose; as well patients with cancer, anoxic brain injury, non-prescription opioid use, or if an accurate medication reconciliation was unable to be obtained. Patients on chronic opioids were compared with those who had not been prescribed opioids in the outpatient setting. The selection criteria and procedure are shown in **Fig 1.**

There was no intervention in this study, as this was on observational study using ICU admission data from an electronic health record. The major outcomes assessed in our study were mortality and hospital length of stay. Mortality was determined at the end of the follow-up period based on the data in MIMIC III. Hospital length of stay was recorded from the date the patient was admitted to the hospital until the date of discharge.

## Statistical analysis

After ICU encounters were obtained from MIMIC-III, a Chi-Square Test was used to determine whether 30-day mortality and 1-year mortality were independent of the use of these medications. As a secondary outcome, we assessed total hospital length of stay (LOS) with the two tailed T-Test. All statistical analyses were performed in R version 4.0.0.

We performed a logistic regression with propensity score weighting to assess both 30-day mortality and 1-year mortality. To assess hospital LOS in days, we built a generalized linear model with an underlying Gaussian distribution and also weighted based on the propensity scores. In each case, the alpha level was 0.05. In the preliminary models, we adjusted the outcomes for chronic opioid use, Sequential Organ Failure Assessment (SOFA) score, age, sex, the number of comorbidities, as well as the disease status for Coronary Artery Disease (CAD), Congestive Heart Failure (CHF), Chronic Obstructive Pulmonary Disease (COPD), Diabetes, End Stage Liver Disease (ESLD), End Stage Renal Disease (ESRD), obesity and stroke. The SOFA score is used to track patients’ status during the stay in an intensive care unit (ICU) to determine the extent of organ dysfunction [12]. The SOFA score is based on six different scores for 6 organ systems: respiratory, cardiovascular, hepatic, coagulation, renal, and neurological. The score has been found useful in predicting the outcome in critically ill patients [13].

To determine whether specific opioids in particular are associated with 30-day mortality, 1-year mortality, and hospital LOS, the same models were used and the covariate for opioid use expanded to adjust for hydromorphone, hydrocodone, oxycodone, morphine, fentanyl, tramadol, methadone, and meperidine.

Lastly, a third set of models was built to determine the association between outcomes and SOFA score, to assess how opioid use is related to mortality after adjusting for improvement in clinical condition. SOFA score between day 1 and day 3 (SOFA D1-D3) represents the improvement in the clinical condition of the admitted patients in each group. This model adjusted for the same variables as the preliminary models along with the effects of each opioid as above.

## Results

A total of 32,384 records were screened. The final sample for the first two sets of models included 22,385 patients with 2,621 (11.7%) patients in the treatment group (mean age 62.37 ± 0.24) and 19,764 (88.3%) in the control group (mean age 61.16 ± 0.60). The demographic distribution of initial patient data is represented in **Table 1**.

**Table 1:** Patient baseline characteristics and bivariate analysis of covariates with respect to opioid use.

Variable	No Opioid Use (n = 19,764)	Opioid Use (n = 2,621)	p-value
Age, years (mean ± SD)	61.16 ± 0.60	62.37 ± 0.24	0.0002
Sex (%F)	48.53%	40.91%	<0.0001
Discharge location home, %	23.27%	29.44%	<0.0001
Length of Stay, days (mean ± SD)	10.02 ± 0.13	11.62 ± 0.43	<0.0001
SOFA Score (mean ± SD)	7.25 ± 0.14	7.41 ± 0.05	0.0353
SOFA D1-D3 (mean ± SD)	3.72 ±0.20	4.00 ± 0.09	0.0118
Number of Comorbidities (mean ± SD)	1.58 ± 0.06	1.57 ± 0.02	0.7826

In regards to demographic distribution data, there are significant differences between those who were prescribed opioids and those who were not prescribed opioids across all the variables except in the number of comorbidities. However, the majority of these differences were not clinically relevant. Clinically relevant differences between opioid and non-opioid users were found in sex, discharge location, length of stay, and comorbidity type.

The comorbidities of interest for this study were coronary artery disease, congestive heart failure (both systolic and diastolic), chronic obstructive pulmonary disease, depression, diabetes mellitus, end stage liver disease (ESLD), end stage renal disease (ESRD), obesity and stroke. There was a statistically significant difference across every comorbidity of interest except for obesity which appears in 3.86% and 3.89% of the non-opioid using and opioid using populations respectively as noted in **Table 2**.

**Table 2:** Bivariate analysis of comorbidities with respect to opioid use.

Variable	No Opioid Use (n = 19,764)	Opioid Use (n = 2,621)	p-value
COPD	4.92%	7.67%	<0.0001
Diabetes	27.89%	30.33%	0.0096
CAD	41.90%	35.14%	<0.0001
CHF	23.32%	26.86%	0.0003
ESRD	3.65%	4.92%	0.0017
ESLD	3.44%	6.10%	<0.0001
Stroke	7.27%	4.54%	<0.0001
Obesity	3.86%	3.89%	0.971
Depression	0.65%	1.37%	<0.0001

COPD, Chronic obstructive pulmonary disease; CAD, coronary artery disease; CHF, congestive heart failure; ESRD, end stage renal disease; ESLD, end stage liver disease.

**Fig 2:**
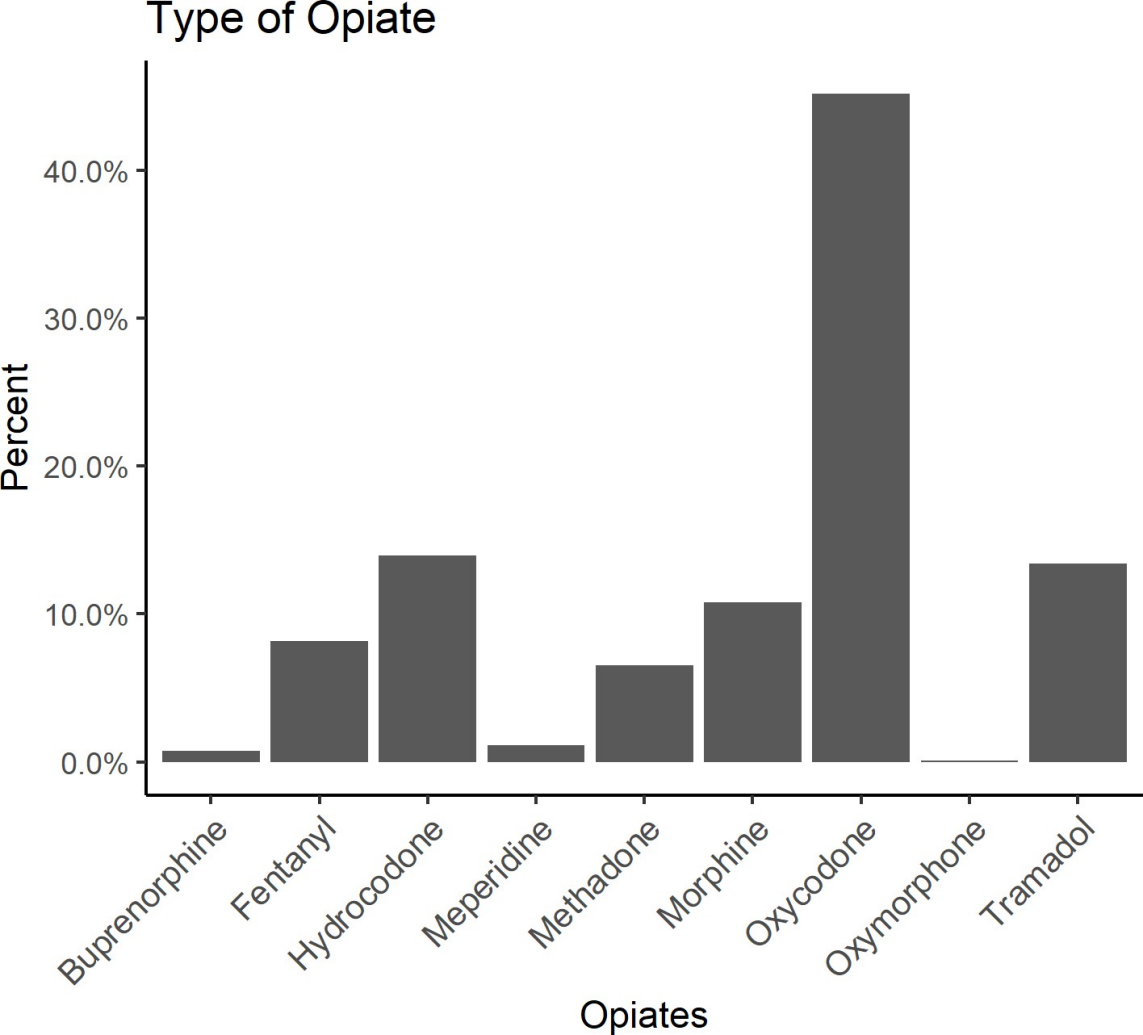
Frequency of opioid prescription by opioid type.

Another feature of interest is the type of opioid prescribed. Among patients with an opioid prescription the most frequently prescribed opioid was oxycodone (45%). There were 3 times as many prescriptions of oxycodone as the next most prescribed opioid, hydrocodone. (**Fig 2**)

Using the preliminary models, it was found that opioid use was associated with increased mortality in both the 30-day and 1-year windows with a respective odds ratio of 1.81 (95% CI, 1.63–2.01; p<0.001) and 1.88 (95% CI, 1.77–1.99; p<0.001). Moreover, patients on opioids had a significantly greater hospital LOS (p<0.001) with a mean hospital stay of 11.62 days (SD = 0.43) versus 10.02 days (SD = 0.13) for patients not on opioids prior to admission. Opioid use was associated with an increased LOS by 1.54 days (95% CI, 1.28–1.81; p <0.001), while controlling for the other covariates. The results of this preliminary model are shown in **Table 3**.

**Table 3:** Preliminary model adjusting for SOFA score for the three outcomes of interest.

	Length of Stay			30-Day Mortality			1-Year Mortality		
Predictors	Estimate	CI	p	Odds Ratio	CI	p	Odds Ratio	CI	p
Opioid Use	1.54	1.28–1.81	<0.001	1.81	1.63–2.01	<0.001	1.88	1.77–1.99	<0.001
Age	-0.04	-0.05 –-0.04	<0.001	1.06	1.06–1.07	<0.001	1.05	1.05–1.06	<0.001
Sex [M]	0.07	-0.20–0.34	0.627	1.27	1.14–1.41	<0.001	1.28	1.20–1.35	<0.001
SOFA Score	0.68	0.66–0.73	<0.001	1.08	1.06–1.09	<0.001	1.03	1.02–1.04	<0.001
# Comorbidities	-0.89	-1.13 –-0.64	<0.001	0.96	0.88–1.05	0.415	0.79	0.75–0.83	<0.001
CAD	0.28	-0.20–0.75	0.252	0.76	0.64–0.90	0.001	0.94	0.85–1.03	0.186
CHF	3.64	3.14–4.13	<0.001	2.00	1.69–2.38	<0.001	2.89	2.62–3.19	<0.001
COPD	0.61	-0.03–1.24	0.064	1.49	1.21–1.82	<0.001	1.59	1.41–1.80	<0.001
Diabetes	0.48	0.065–0.89	0.023	1.17	1.01–1.36	0.034	1.64	1.50–1.78	<0.001
ESLD	2.92	2.19–3.65	<0.001	4.53	3.68–5.54	<0.001	5.06	4.43–5.76	<0.001
ESRD	3.00	2.25–3.75	<0.001	1.18	0.93–1.50	0.174	2.59	2.27–2.96	<0.001
Obesity	0.20	-0.51–0.92	0.577	0.30	0.18–0.47	<0.001	0.38	0.30–0.48	<0.001
Stroke	2.23	1.64–2.82	<0.001	1.30	1.05–1.60	0.015	1.36	1.20–1.53	<0.001
Observations	22385			22385			22385		
Tjur’s R^2^	1.00			0.035			0.099		

A second set of models was created to account for the specific type of opioid. In these models, nearly all of the opioids showed a statistically significant change in their 30-day or 1-year mortality **S1 Table**. In addition, those who use fentanyl have a longer length of stay by 3.42 days (95% CI, 2.79–4.06) compared to those who did not use fentanyl.

Using the second set of models for 30-day mortality, 1-year mortality, and hospital length of stay, it was found that patients with obesity have a 71% (95% CI, 0.17–0.46) and 62% (95% CI, 0.30–0.48) decrease in the risk of 30-day and 1-year mortality, respectively. In addition, people with ESLD have 4.62 (95% CI, 3.75–5.67) and 5.10 (95% CI, 4.47–5.81) times the risk of 30-day and 1-year mortality, respectively. Lastly, a one unit increase in SOFA scores is associated with an 8% (95% CI, 1.06–1.09) increase in 30-day mortality and a 3% (95% CI, 1.02–1.04) increase in 1-year mortality while adjusting for the other covariates.

For the third set of models that adjusted for SOFA D1-D3 as well as the other covariates, there were a total of 8,711 total participants for whom a SOFA D1-D3 score could be calculated. Of these, 7,598 (87.2%) were in the non-opioid using group and 1,113 (12.8%) were in the opioid using group. The results showed that opioid use was associated with increased mortality in both the 30-day and 1-year windows with a respective odds ratio of 1.84 (95% CI, 1.60–2.11; p<0.001) and 1.75 (95% CI, 1.62–1.91; p<0.001) **S2 Table**. In addition, those who used meperidine spent 5.93 more days in the hospital (95% CI,2.31–9.54) compared to non-meperidine users. An association of a decreased risk of 30-day mortality for those with CAD was also noted.

## Discussion

In this study, we report that even after adjusting for severity of illness, chronic opioid users have worse outcomes in the ICU compared to those not on chronic opioids. Chronic opioid usage was associated with increased hospital length of stay and increased mortality at both 30 days and 1 year after ICU admission. All models had comparable rates of increased 30-day and 1-year mortality rates with respect to SOFA score with highest risk at 1 year implying the long-term effects of critical illness and possible role of chronic opioids. This study highlights the relationship between chronic opioid use and clinical outcomes in critical care settings, to help equip providers with information when engaging in risks-benefit discussions with patients.

Similar findings were demonstrated in a study [4] where both the occasional opioid use and chronic opioid therapy groups were more likely to experience 30-day hospital readmission in a sample of acute medical admissions, a relationship that remained consistent across the partially and fully adjusted models. In a subset of patients undergoing surgery, it has been shown that preoperative opioid use is an independent risk factor for longer length of stay, higher 30-day readmission rates and probability of being discharged to a rehabilitation facility, and greater costs in the postoperative period [14].

One explanation for increased hospital stay and mortality among chronic opioid users is the potential for greater infectious complications with chronic opioid abuse. Studies have shown that opioid abuse causes immunosuppression by disrupting both innate and adaptive components of the immune system [15, 16]. Opioid abuse is a critical risk factor for increased susceptibility and severity of bacterial infection, including S. pneumoniae [17, 18]. It was also demonstrated that morphine treatment leads to this effect via a diminished release of interleukins and decreased neutrophil recruitment [19]. These mechanisms could be extrapolated to our population subset which included patients with chronic prescription opioid use as opposed to chronic nonprescription opioid abuse.

Opioids are also known to have central adverse effects of oversedation, respiratory depression, and cough suppression [20]. These effects may also predispose patients to aspiration, especially when used in larger than usual doses in a patient with chronic opioid induced hyperalgesia. Need for greater opioid doses acutely may also play a role in increased length of stay, by affecting time to extubation [21]. Conversely, underdosing equivalent opioid analgesics when shorter acting agents are used during transitions of care may precipitate withdrawal and hemodynamic instability associated with the same. One observational (uncontrolled) study in ICU patients suggests that opioid withdrawal might play a role in immunosuppression [22].

Hydrocodone, oxycodone, and tramadol are the most commonly prescribed outpatient opioids in the USA. Our dataset showed the oxycodone was the most prescribed, followed by hydrocodone and Tramadol. While historically, hydrocodone was the most prescribed opioid in the outpatient settings, the amounts of oxycodone dispensed from retail pharmacies in the U.S. increased by 678% between 1997 and 2006. That increase, along with the known geographical variation in opioid prescription [23], is likely to be the reason why oxycodone was the most reported opioid as outpatient.

It is unclear by what mechanism this effect is generated, whether baseline functional status, modestly poorer in patients on long term opioids [24], and chronic illness severity are playing a role.

### Limitations of the study

We attempted to address the effect of confounding variables by extensively adjusting for presence and type of opioid use, demographic differences, differences in illness severity and comorbidities as described in the statistical analysis section, but we have not accounted for a the possible impact of opioid tolerance, difference in dose, chronic use of other neuroleptics or other hidden confounding variables. The dataset, while providing a comprehensive list of home medications, does not capture the dose and duration of using these medications. Regardless, our study still shows a qualitative effect of chronic opiate use regardless of dose and duration of therapy.

There is also the concern of this study being a product of a single database and of patients with an average SOFA score of 7, which may limit its generalizability to other centers and sicker populations. We hope these questions can be answered by future studies.

Lastly, there is the concern of coding bias from using electronic health records. Studies using electronic health records share many common limitations such as coding errors, inaccurate reporting of past medical history, and underdiagnosis [25]. However, we feel that these errors and limitations are universally applied to all the dataset, which cancels out any bias that could have been introduced to the two studied groups.

## Conclusions

This study is unique in its attempt to explore the relationship between chronic opioid use and clinical outcomes in critical care settings. While outpatient opioid use has been decreasing over the last decade, the US has witnessed 153,260,450 opioids prescriptions in 2019, with 46.7 Opioid Dispensing Rate Per 100 Persons [26]. The high utilization rate necessitates a deeper look at the healthcare sequelae of chronic opioid usage, and this makes the findings in this study insightful to primary care providers, who are the primary prescribers of chronic opioids.

The findings of this study are that chronic opioid usage was associated with a statistically significant increased hospital and ICU length of stay, as well as increased mortality at both 30 days and 1 year after an ICU admission after controlling for age, sex, delta sofa, the number of comorbidities, presence of CAD, CHF, COPD, diabetes, ESLD, ESRD, obesity and stroke. This is key information when making a risk-benefit decision when prescribing opioids for chronic usage.

## Supporting information

S1 Table Logistic regression adjusting for individual opioids 30-day and 1-year mortality. (DOCX)

S2 Table Logistic regression adjusting for opioid index 30-day and 1-year mortality with SOFA D1-D3 score. (DOCX)

S3 Table Logistic regression adjusting for individual opioids: 30-day and 1-year mortality with SOFA D1-D3 score. (DOCX)
