# Supplementary material for: Association of prescription opioid use on mortality and hospital length of stay in the intensive care unit
Source: PLoS One. 2021 Apr 22;16(4):e0250320. doi: 10.1371/journal.pone.0250320 (PMC8061930; doi:10.1371/journal.pone.0250320)
Supplement: S1 Table — (DOCX) [file pone.0250320.s001.docx]

**S1 Table. Logistic regression adjusting for individual opioids 30-day and 1-year mortality.**

|  | **1-year mortality** | | | **30-day mortality** | | |
| --- | --- | --- | --- | --- | --- | --- |
| *Predictors* | *Odds Ratios* | *CI* | *p* | *Odds Ratios* | *CI* | *p* |
| (Intercept) | 0.00 | 0.00 – 0.00 | **<0.001** | 0.00 | 0.00 – 0.00 | **<0.001** |
| Age | 1.05 | 1.05 – 1.06 | **<0.001** | 1.06 | 1.06 – 1.07 | **<0.001** |
| Gender [M] | 1.27 | 1.20 – 1.35 | **<0.001** | 1.23 | 1.11 – 1.37 | **<0.001** |
| SOFA | 1.03 | 1.02 – 1.04 | **<0.001** | 1.08 | 1.06 – 1.09 | **<0.001** |
| # comorbidities | 0.79 | 0.75 – 0.83 | **<0.001** | 0.98 | 0.90 – 1.07 | 0.712 |
| CAD | 0.93 | 0.85 – 1.03 | 0.162 | 0.75 | 0.63 – 0.88 | **0.001** |
| CHF | 2.83 | 2.56 – 3.13 | **<0.001** | 1.88 | 1.58 – 2.23 | **<0.001** |
| COPD | 1.56 | 1.37 – 1.76 | **<0.001** | 1.46 | 1.19 – 1.79 | **<0.001** |
| Diabetes | 1.61 | 1.47 – 1.75 | **<0.001** | 1.15 | 0.99 – 1.33 | 0.067 |
| ESLD | 5.10 | 4.47 – 5.81 | **<0.001** | 4.62 | 3.75 – 5.67 | **<0.001** |
| ESRD | 2.65 | 2.32 – 3.03 | **<0.001** | 1.25 | 0.98 – 1.59 | 0.065 |
| Obesity | 0.38 | 0.30 – 0.48 | **<0.001** | 0.29 | 0.17 – 0.46 | **<0.001** |
| Stroke | 1.33 | 1.17 – 1.50 | **<0.001** | 1.26 | 1.01 – 1.55 | **0.034** |
| Hydromorphone | 0.95 | 0.73 – 1.23 | 0.721 | 1.03 | 0.63 – 1.59 | 0.886 |
| Hydrocodone | 1.16 | 1.04 – 1.29 | **0.008** | 0.85 | 0.67 – 1.05 | 0.140 |
| Oxycodone | 1.80 | 1.68 – 1.92 | **<0.001** | 1.66 | 1.48 – 1.86 | **<0.001** |
| Morphine | 2.13 | 1.92 – 2.35 | **<0.001** | 2.70 | 2.31 – 3.14 | **<0.001** |
| Fentanyl | 1.86 | 1.66 – 2.10 | **<0.001** | 2.18 | 1.81 – 2.61 | **<0.001** |
| Tramadol | 1.45 | 1.31 – 1.60 | **<0.001** | 1.11 | 0.91 – 1.33 | 0.287 |
| Methadone | 1.49 | 1.26 – 1.76 | **<0.001** | 1.51 | 1.10 – 2.04 | **0.008** |
| Meperidine | 0.29 | 0.14 – 0.54 | **<0.001** | 1.39 | 0.65 – 2.60 | 0.352 |
| Observations | 22385 | | | 22385 | | |
| R^2^ Tjur | 0.102 | | | 0.038 | | |
