# Supplementary material for: Association of prescription opioid use on mortality and hospital length of stay in the intensive care unit
Source: PLoS One. 2021 Apr 22;16(4):e0250320. doi: 10.1371/journal.pone.0250320 (PMC8061930; doi:10.1371/journal.pone.0250320)
Supplement: S2 Table — (DOCX) [file pone.0250320.s002.docx]

**S2 Table. Logistic regression adjusting for opioid index 30-day and 1-year mortality with SOFA D1-D3 score.**

|  | **1-year mortality** | | | **30-day mortality** | | |
| --- | --- | --- | --- | --- | --- | --- |
| *Predictors* | *Odds Ratios* | *CI* | *p* | *Odds Ratios* | *CI* | *p* |
| (Intercept) | 0.00 | 0.00 – 0.00 | **<0.001** | 0.00 | 0.00 – 0.00 | **<0.001** |
| Age | 1.06 | 1.05 – 1.06 | **<0.001** | 1.06 | 1.06 – 1.07 | **<0.001** |
| Gender [M] | 1.32 | 1.21 – 1.43 | **<0.001** | 1.35 | 1.17 – 1.55 | **<0.001** |
| SOFA | 0.97 | 0.96 – 0.98 | **<0.001** | 0.98 | 0.96 – 1.00 | **0.021** |
| # comorbidities | 0.81 | 0.76 – 0.87 | **<0.001** | 1.13 | 1.02 – 1.25 | **0.018** |
| CAD | 0.95 | 0.83 – 1.09 | 0.457 | 0.65 | 0.52 – 0.80 | **<0.001** |
| CHF | 2.25 | 1.97 – 2.58 | **<0.001** | 1.18 | 0.95 – 1.47 | 0.125 |
| COPD | 1.47 | 1.24 – 1.74 | **<0.001** | 1.17 | 0.90 – 1.52 | 0.235 |
| Diabetes | 1.41 | 1.25 – 1.59 | **<0.001** | 0.82 | 0.68 – 1.00 | 0.050 |
| ESLD | 3.51 | 2.91 – 4.23 | **<0.001** | 3.31 | 2.50 – 4.34 | **<0.001** |
| ESRD | 2.35 | 1.95 – 2.84 | **<0.001** | 0.94 | 0.69 – 1.27 | 0.712 |
| Obesity | 0.43 | 0.31 – 0.58 | **<0.001** | 0.14 | 0.05 – 0.30 | **<0.001** |
| Stroke | 1.30 | 1.10 – 1.53 | **0.002** | 1.40 | 1.09 – 1.78 | **0.007** |
| Opiates | 1.75 | 1.62 – 1.91 | **<0.001** | 1.84 | 1.60 – 2.11 | **<0.001** |
| Observations | 8711 | | | 8711 | | |
| R^2^ Tjur | 0.103 | | | 0.038 | | |
