# Supplementary material for: Association of prescription opioid use on mortality and hospital length of stay in the intensive care unit
Source: PLoS One. 2021 Apr 22;16(4):e0250320. doi: 10.1371/journal.pone.0250320 (PMC8061930; doi:10.1371/journal.pone.0250320)
Supplement: S3 Table — (DOCX) [file pone.0250320.s003.docx]

**S3 Table. Logistic regression adjusting for individual opioids: 30-day and 1-year mortality with SOFA D1-D3 score.**

|  |  | **1-year mortality** | | | **30-day mortality** | | |
| --- | --- | --- | --- | --- | --- | --- | --- |
|  | *Predictors* | *Odds Ratios* | *CI* | *p* | *Odds Ratios* | *CI* | *p* |
|  | (Intercept) | 0.00 | 0.00 – 0.00 | **<0.001** | 0.00 | 0.00 – 0.00 | **<0.001** |
|  | Age | 1.06 | 1.05 – 1.06 | **<0.001** | 1.07 | 1.06 – 1.07 | **<0.001** |
|  | Gender [M] | 1.30 | 1.19 – 1.41 | **<0.001** | 1.28 | 1.11 – 1.47 | **0.001** |
|  | SOFA | 0.97 | 0.96 – 0.98 | **<0.001** | 0.98 | 0.96 – 1.00 | **0.023** |
|  | # comorbidities | 0.82 | 0.77 – 0.88 | **<0.001** | 1.15 | 1.04 – 1.27 | **0.006** |
|  | CAD | 0.94 | 0.82 – 1.07 | 0.369 | 0.64 | 0.52 – 0.80 | **<0.001** |
|  | CHF | 2.19 | 1.91 – 2.50 | **<0.001** | 1.09 | 0.87 – 1.35 | 0.458 |
|  | COPD | 1.43 | 1.21 – 1.69 | **<0.001** | 1.15 | 0.88 – 1.49 | 0.299 |
|  | Diabetes | 1.37 | 1.22 – 1.54 | **<0.001** | 0.80 | 0.66 – 0.98 | **0.027** |
|  | ESLD | 3.64 | 3.02 – 4.39 | **<0.001** | 3.60 | 2.72 – 4.73 | **<0.001** |
|  | ESRD | 2.41 | 2.00 – 2.91 | **<0.001** | 1.01 | 0.74 – 1.37 | 0.939 |
|  | Obesity | 0.42 | 0.30 – 0.56 | **<0.001** | 0.13 | 0.05 – 0.28 | **<0.001** |
|  | Stroke | 1.27 | 1.07 – 1.49 | **0.005** | 1.35 | 1.06 – 1.73 | **0.015** |
|  | Hydromorphone | 0.90 | 0.56 – 1.41 | 0.665 | 0.60 | 0.20 – 1.39 | 0.294 |
|  | Hydrocodone | 1.20 | 1.04 – 1.39 | **0.014** | 1.09 | 0.84 – 1.40 | 0.512 |
|  | Oxycodone | 1.56 | 1.41 – 1.71 | **<0.001** | 1.42 | 1.21 – 1.66 | **<0.001** |
|  | Morphine | 1.96 | 1.71 – 2.24 | **<0.001** | 2.82 | 2.33 – 3.40 | **<0.001** |
|  | Fentanyl | 1.78 | 1.52 – 2.09 | **<0.001** | 2.29 | 1.81 – 2.86 | **<0.001** |
|  | Tramadol | 1.33 | 1.14 – 1.54 | **<0.001** | 1.12 | 0.86 – 1.43 | 0.381 |
|  | Methadone | 1.24 | 0.95 – 1.60 | 0.104 | 1.80 | 1.16 – 2.67 | **0.005** |
|  | Meperidine | 0.63 | 0.27 – 1.29 | 0.236 | 3.37 | 1.46 – 6.95 | **0.002** |
|  | Observations | 8711 | | | 8711 | | |
|  | R^2^ Tjur | 0.106 | | | 0.042 | | |
